# Yield Response and Nutrient Use Efficiencies under Different Fertilizer Applications in Maize (*Zea mays* L.) In Contrasting Agro Ecosystems

**DOI:** 10.9734/IJPSS/2019/v29i330141

**Published:** 2019-08-16

**Authors:** Tesfaye Balemi, Jairos Rurinda, Mesfin Kebede, James Mutegi, Gebresilasie Hailu, Tolcha Tufa, Tolera Abera, Tesfaye Shiferaw Sida

**Affiliations:** 1 Ethiopian Institute of Agricultural Research, P.O.Box. 2003, Addis Ababa, Ethiopia; 2 International Plant Nutrition Institute (IPNI), C/O IFDC – East and Southern Africa Division, ICIPE Compound, Duduville – Kasarani, Thika Road, P.O.Box 30772-00100, Nairobi, Kenya; 3 ILRI/CIMMYT, Gurd Shola, P.O.Box. 5689, Addis Ababa, Ethiopia

**Keywords:** Ethiopia, Zea mays L, nutrient omission trials, agronomic efficiency, apparent recovery efficiency, nutrient uptake

## Abstract

Variability in crop response and nutrient use efficiencies to fertilizer application is quite common under varying soil and climatic conditions. Understanding such variability is vital to develop farm- and area- specific soil nutrient management and fertilizer recommendations. Hence the objectives of this study were to assess maize grain yield response to nutrient applications for identifying yield-limiting nutrients and to understand the magnitude of nutrient use efficiencies under varying soil and rainfall conditions. A total of 150 on-farm nutrient omission trials (NOTs) were conducted on farmers’ field in high rainfall and moisture stress areas. The treatments were control, PK, NK, NP, NPK and NPK+ secondary and micronutrients. Maize grain yield, nutrient uptake, agronomic and recovery efficiencies of N and P differed between fertilizer treatments and between the contrasting agro-ecologies. The AE_N_ ranged from 24.8 to 32.5 kg grain kg^-1^ N in Jimma area and from 1.0 kg grain kg^-1^ N (NK treatment) to 10.2 kg grain kg^-1^ N (NPK treatment) at Adami Tullu and from 0.1 kg grain kg^-1^ N (NK treatment) to 8.3 kg grain kg^-1^ N (NPK treatment) at Bulbula. The differing parameters between the agro-ecologies were related to difference in rainfall amount and not to soil factors. Grain yield response to N application and agronomic efficiencies of N and P were higher in the high rainfall area than in the moisture stress areas. Grain yield responded the most to nitrogen (N) application than to any other nutrients at most of the experimental sites. Owing to the magnificent yield response to N fertilizer in the current study, proper management of nitrogen is very essential for intensification of maize productivity in most maize growing areas of Ethiopia.

Peer-review history: The peer review history for this paper can be accessed here: http://www.sdiarticle3.com/review-history/49748

## ABBREVIATIONS

AE_N_ : Agronomic efficiency of applied N

AE_P_, : Agronomic efficiency of applied P

ARE_N_: Apparent recovery efficiency of applied N

ARE_P_: Apparent recovery efficiency of applied P

## 1. INTRODUCTION

Food insecurity is a great concern in Sub-Saharan Africa (SSA) given the ever-increasing human population, changing climate and persistently low crop yields. This is particularly so in Ethiopia, which is the second most populous country in Africa with an average annual population growth rate of 2.7%. Maize (*Zea mays* L.) has increasingly become one of the most important staple food crops in Ethiopia. Its production and consumption have grown widely across many regions. However, the current average maize yield is 3944 kg ha^-1^ [1], which is much lower than its yield potential. One of the major reasons for the low maize productivity in SSA and in Ethiopia in particular is poor soil nutrients status. Nitrogen (N) and phosphorus (P) were specifically deficient in most parts of the country [2,3]. The wider variability in soil fertility, climate and farmers nutrient management practices further contributed to low maize productivity at national level. Farmers in the maize growing regions apply small amounts of fertilizers containing mainly N and P [4] Moreover, the recovery fractions of the applied nutrients are often quite low due to nutrient losses, unbalanced nutrient application [5] and in some regions due to limited soil moisture [6]. Moreover, poor crop and nutrient management practices such as lack of weeding, low plant density and use of inappropriate blanket fertilizer recommendations can also reduce nutrient use efficiency and crop yields [7,8].

In Ethiopia, regional fertilizer recommendations have been developed for maize [9], which is slightly region specific than the earlier single blanket recommendation of 100 kg DAP and 200 kg Urea ha^-1^. Yet cropping systems, crop management practices, soil types and fertility status, climatic conditions and other factors governing yield response to nutrients, vary considerably in space and time [10,11]. Due to such localized differences in crop growing conditions and the soils’ indigenous nutrient supply capacity, grain yield response to fertilizer application as well as nutrient use efficiencies could vary across the maize production regions of the country as reported by Kihara et al. [10] in many Sub-Sahara Africa countries, by Kurwakumire et al. [11] in Zimbabwe and by Wakene et al. [9] in Ethiopia. Both blanket and regional fertilizer recommendations often lead to either over-fertilization or under-fertilization by individual farmers. Excessive application, especially of N and P fertilizers, may result in loss of investment in fertilizer input, nutrient accumulation in the soil (low nutrient use efficiency) and environmental pollution [12]. By contrast, under-fertilization may lead to nutrient mining owing to the imbalance between nutrient removed by the crop and the nutrient applied in the form of fertilizer. To increase nutrient use efficiencies, minimize soil degradation and sustain intensification of crop productivity, more site-specific nutrient management options are recommended, especially for SSA where the cropping systems are highly heterogeneous [10, 13]. Several studies revealed that optimum N and P rates differed for different maize growing locations [9,14] and with different cropping system [15], suggesting that the old tradition of using blanket fertilizer recommendation can no more be an appropriate practice to follow. Other studies confirm that ignoring important soils nutrients, other than N and P in any crop production in the country could result in significant grain yield losses at least in specific locations [16,17,18] and hence need to be carefully handled.

To develop strategies for improved nutrient management and optimize fertilizer recommendations in specific regions, there is a need to understand the nutrient status of the soil, the magnitude of crop response to fertilizer applications and the nutrient use efficiencies in a particular location/region.

The objectives of this study were to: (1) assess maize grain yield response to different nutrients, (2) identify yield limiting nutrients and (3) understand the magnitude of agronomic and apparent fertilizer recovery efficiencies under variable soil and rainfall conditions.

## 2. MATERIALS AND METHODS

### 2.1 Study Sites

**Fig. 1:**
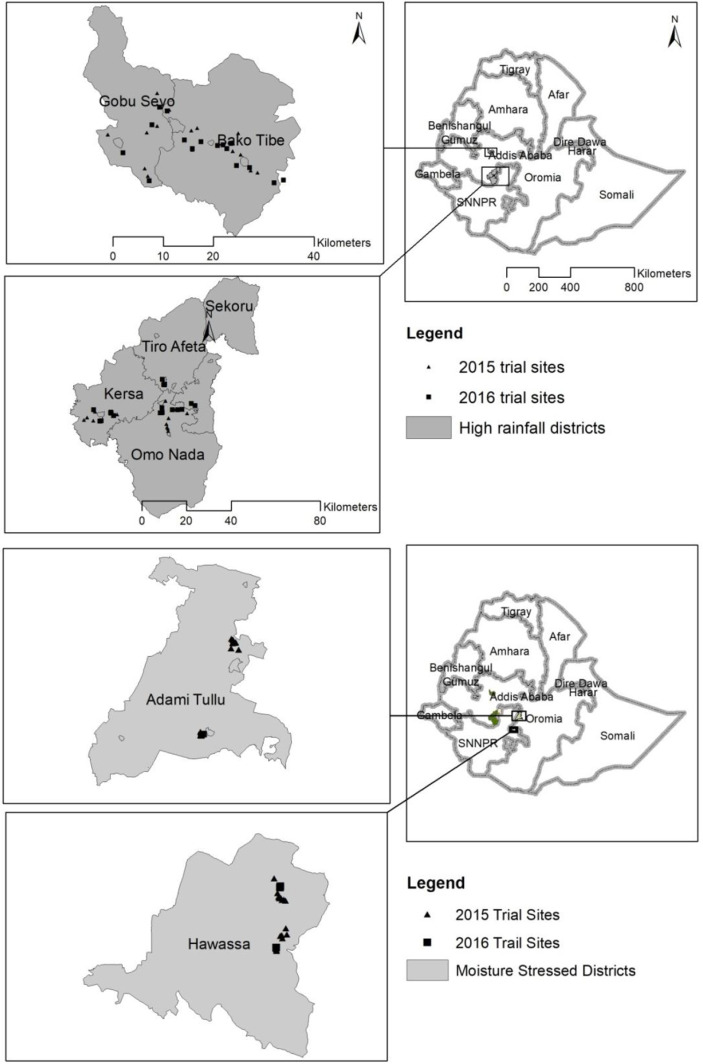
Multi-location nutrient omission trial (NOTs) study sites located across major maize production areas in contrasting agro ecological zones in Ethiopia

**Fig. 2:**
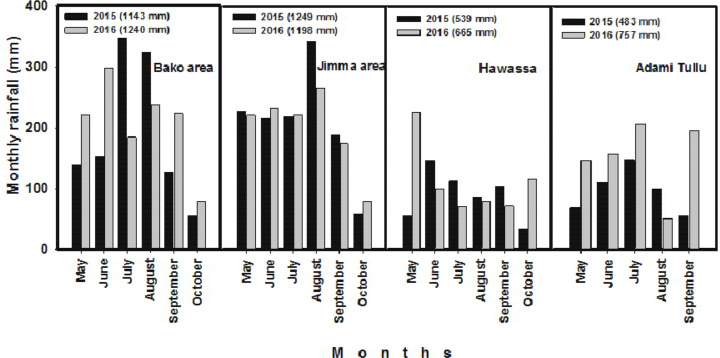
Crop growing season monthly rainfall (mm) received in the nutrient omission studied sites in major maize production areas in Ethiopia

Nutrient Omission Trials (NOTs) were conducted on farmers’ fields in major maize production areas of Ethiopia over two cropping seasons, 2015 and 2016. The NOTs study sites were purposefully selected to cover a broad range of major maize growing areas in Ethiopia, representing both high rainfall and moisture stress agro-ecologies. Selection of the study sites were guided by soil and climate maps, and the African Soil Information System (AfSIS) crop mask to classify major maize production areas in terms of 1 km pixel resolution [19]. Fields with gentle slopes, minimum soil heterogeneity and that were large enough to accommodate six treatments (described subsequently) were selected for the establishment of the NOTs. A total of 150 nutrient omission trials (*N* = 88 in 2015 and *N* = 62 in 2016) were established across eight districts: Hawassa, Adami Tullu/Bulbula, Bako Tibe, Gobu Sayo, Omo Nada, Kersa, Tiro Afeta and Sekoru (Fig. 1). Adami Tullu/Bulbula and Hawassa are characterized as semi-arid moisture stress areas while the rest of the districts, hereafter described as Bako and Jimma areas for 2016 season summary data, are characterized as high rainfall sub-humid areas. The total monthly rainfall of all the experimental sites during the two cropping seasons is presented in Fig. 2. The soils in Adami Tullu/Bulbula and Hawassa are sandy loam dominated by andosol with neutral soil pH whereas the soils in Bako and Jimma areas are generally clay dominated by reddish or reddish brown nitisols with acidic soil pH (Table 1).

**Table 1:** pH, organic carbon (OC), total nitrogen (TN), available phosphorus (P) and exch. potassium (K), Calcium (Ca), magnesium (Mg), zinc (Zn), copper (Cu), manganese (Mn) and iron (Fe) contents of soils in the different nutrient omission experimental sites

Soil parameters	Experimental locations						
	Bako Tibe	Gobu Sayo	Omo Nada	Kersa	Adami Tullu	Bulbula	Hawassa
pH (H2O)	4.6-5.8 (5.1)	4.7-5.8 (5.1)	4.6-5.6 (5.1)	4.5-6.0 (5.1)	6.8-7.9 (7.2)	6.7-7.5 (7.2)	6.7-7.4 (7.1)
OC (%)	1.3-2.7 (2.2)	2.0-2.9 (2.3)	0.8-2.4 (1.6)	1.0-2.1 (1.7)	0.6-1.1 (0.8)	0.6-0.9 (0.7)	0.4-0.7 (0.6)
TN (%)	0.13-0.23(0.19)	0.19-0.29(0.23)	0.12-0.27(0.17)	0.11-0.23(0.19)	0.05-0.13(0.09)	0.09-0.10(0.10)	0.03-0.08(0.05)
Available P (mg kg-1)	3.9-61.8 (11.5)	5.5-10.6 (7.7)	5.5-42.7 (17.0)	4.3-19 (11.1)	11.9-61.4(26.0)	26.2-56.2(41.5)	18.2-55.7(31.0)
Exch. K (mg kg-1)	49-1488 (514)	133-868 (541)	249-716 (514)	379-771 (556)	127-564 (276)	116-222 (144)	50-228 (146)
Ca (g kg-1)	1.6-5.3(3.6)	1.8-4.0(3.2)	2.2-3.9(2.7)	1.4-5.3(2.8)	0.9-4.6(2.3)	0.99-2.5(1.6)	0.77-2.5(1.9)
Mg (g kg-1)	0.4-1.5(0.9)	0.6-1.1(0.9)	0.45-1.1(0.67)	0.3-1.5(0.7)	0.12-0.47(0.25)	0.17-0.36(0.26)	0.08-0.28(0.16)
Zn (ppm)	2-16(8)	3-7(5)	5-19(13)	4-18(8)	0.5-1.6(1.0)	0.6-1.0(0.8)	0.4-0.9(0.6)
Cu (ppm)	1-4(3)	2.9-5.8(4.8)	1-9(5)	7-26(11)	15.7-41.4(24.4)	13.5-20.1(16.0)	20.0-27.7(22.3)
Mn (ppm)	29-115(66)	58-98(87)	64-261(142)	73-231(149)	45-130(84)	68-106(88)	61-112(79)
Fe (ppm)	44-189 (92)	50-113(75)	145-233(177)	69-189(137)	0.03-0.1(0.06)	0.02-0.06(0.04)	0.02-0.04(0.024)

Values are ranges and those in parentheses are mean

### 2.2 Nutrient Omission Trials Set Up and Management

The nutrient omission trials (NOTs) consisted of six treatments that included: the control, PK (0-40-40), NK (120-0-40), NP (120-40-0) NPK (120-40-40) in kg ha^-1^ and NPK + secondary nutrients such as sulphur (S), calcium (Ca), magnesium (Mg) + micro-nutrients such as boron (B) and zinc (Zn), which here after is denoted as NPK+ (Table 3). The rates of each nutrient in the last treatment were 120-40-40-20-10-10-5-5 (kg ha^-1^) in that order. The treatments were replicated across individual farmers’ fields. To understand the temporal variability of yield response to fertilizer application, the NOTs were repeated in 2016 cropping season in the same fields used for 2015 season, using different new plots to avoid confounding effects of residual nutrients.

The experimental fields for all NOTs were prepared with an oxen-drawn mouldboard plough. The plot sizes of each treatment were 8 m × 8 m (64 m^2^), and a hybrid maize variety recommended for each area was used as a test crop. In Jimma and Bako areas, a hybrid variety, BH661 (with 160 average days to maturity) was used. In Hawassa and Adami Tullu/Bulbula areas a hybrid variety, BH540 (with 145 average days to maturity) was used. Plant spacing of 75 cm (inter-row) × 25 cm (intra-row) was used in order to maintain a plant population of 53,000 plants ha^-1^. In each area, the planting time was adjusted to match farmers planting windows. All nutrients were applied at planting except N, which was applied in three equal splits, 1/3 at planting, 1/3 at V6 (21 days after planting, DAP), and 1/3 at V10 (35 DAP). Urea, triple super phosphate (TSP), murate of potash (MOP), hydrated forms of magnesium, calcium and zinc sulphates and borax were used as fertilizer sources for N, P, K, Mg, Ca, Zn and B, respectively. Nutrient application rates were assumed to be non-limiting at each site. The trials were uniformly managed by researchers for weeds, diseases and pests using appropriate control measures.

### 2.3 Soil and Plant Analyses

Soil samples were collected from a depth of 0-20 cm at trial establishment before the application of fertilizers. Soil samples were obtained from four points in each experimental field based on a Y-frame methodology, and the four samples collected in each field were thoroughly mixed to form a composite sample. The composite soil samples were analyzed at IITA Laboratory in Ibadan, Nigeria for major soil properties. The soil properties analyzed included soil organic carbon (OC) using chromic acid digestion [20], Total N using Kjeldahl digestion [21], soil pH (1:2.5 soil: water suspension) according to [22], available P, Exchangeable cations and micronutrients (Zn, Cu, Mn, and Fe) all of which were determined using Mehlich 3 extraction procedure [23].

At harvest the stover and grain samples were oven dried and ground for N and P analyses. Phosphorus was determined using ascorbic acid method following a procedure described by Murphy and Rilly [24], while N was determined after digesting the plant samples with sulphuric acid following a procedure described by Novozamsky et al. [25].

### 2.4 Determination of Grain Yield and Nutrient Use Efficiencies

Harvesting was done at physiological maturity from a net plot area of 4 m × 4.5 m (18 m^2^). The field grain weight was recorded and the grain yield was determined after adjusted to the standard 12.5% moisture content. Agronomic efficiency and apparent fertilizer recovery efficiency were determined using the following formulas according to Fageria et al. [26]:

Agronomic efficiency (AE) was determined as:

$$AE=\frac{GYt-GYc}{Na}$$ 1

Where, *GYt* is the grain yield of fertilizer treated plot (kg), *GYc* is the grain yield of the fertilizer untreated plot (kg) and *Na* is the amount of nutrient applied (kg).

Apparent fertilizer recovery efficiency (ARE) was determined as:

$$ARE=\frac{NUt-NUc}{Na}\ldots$$ 2

Where, *NUt* is the nutrient uptake (in grain and straw) of the fertilizer treated plot and *NUc* is the nutrient uptake (in grain and straw) of fertilizer untreated plot.

### 2.5 Data Analysis

The effects of fertilizer treatments on maize grain yield, nutrient uptake, agronomic and apparent recovery efficiencies were analyzed using analysis of variance (ANOVA) procedures using a Statistical Analysis System (SAS), version 9.3 Software,(SAS institute INC., Cary, USA). The ANOVA was computed based on PROC GLM procedure and when ANOVA showed the presence of significant treatment effects, mean separation was carried out using Tukey's test at α=5% level of significance.

## 3. RESULTS

### 3.1 Grain Yield at Different Locations of the Two Agro-ecologies

**Fig. 3:**
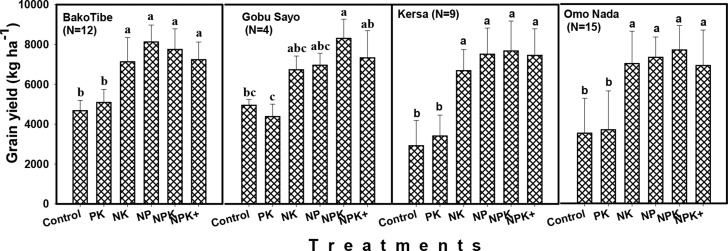
Effects of fertilizer treatments on responsive soils at Bako Tibe, Gobu Sayo, Kersa

Grain yield generally tended to be higher at the high rainfall areas than at the moisture stress areas, especially during 2015 season. At the high rainfall areas, grain yield ranged from 2916 kg ha^-1^ for control treatment at Kersa to 8301 kg ha^-1^ for NPK treatment at Gobu Sayo during the same year (Fig. 3). At the moisture stress areas, however, it ranged between 1061 kg ha^-1^ for NK treatment at Bulbula to 5925 kg ha^-1^ for NPK+ treatment at Hawassa (Fig. 5A). During 2016 season, grain yield in the high rainfall areas ranged from 1434 kg ha^-1^ for the control treatment to 7796 kg ha^-1^ for the NPK treatment in Jimma area. Grain yield during the same year ranged from 1787 kg ha^-1^ to 6928 kg ha^-1^, for the control and NPK+ treatments at Adami Tullu, in the moisture stress area (Fig. 5B).

**Fig. 4:**
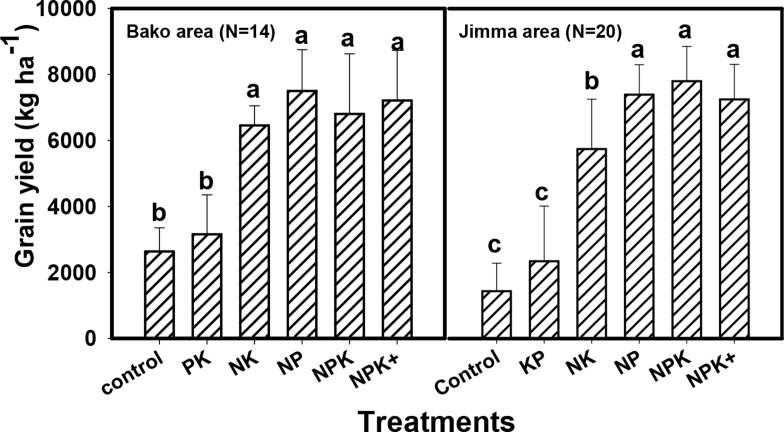
Effects of fertilizer treatments on responsive soils in Bako (Three districts) and Jimma areas (Four districts) during 2016 cropping season (Bars followed by the same letter for the same location are not significantly different)

**Fig. 5:**
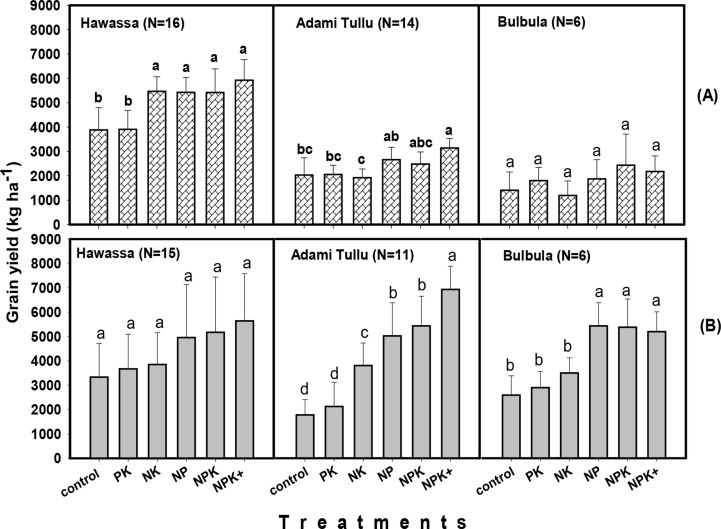
Effects of fertilizer treatments on maize grain yield at Hawassa, Adami Tullu and Bulbula in 2015 (A) and 2016 (B) cropping seasons. (Bars followed by the same letter for the same site are not significantly different)

For high rainfall areas, grain yields obtained from NP, NPK and NPK+ were consistent between seasons. By contrast, grain yields from control, N and P omitted treatments were lower in 2016 compared with 2015. In the moisture stress areas, particularly at Adami Tullu and Bulbula, grain yield were higher in 2016 cropping season than in 2015 season, which was characterized by erratic rainfall (Fig. 5).

### 3.2 Maize Yield Response to N

There was a wide spatial and temporal variability in maize yield response to nutrients across the study sites. Maize grain yield responded drastically to nitrogen (N) application almost at all the study sites. The magnitude of the response to N application was, however, much higher for the high rainfall than low rainfall/moisture stress areas. The grain yield response ranged from 2657 to 4266 kg ha^-1^ in 2015 and from 3648 to 5454 kg ha^-1^ in 2016 in high rainfall areas, while it ranged from 383 to 1513 kg ha^-1^ in 2015 and from 1500 to 3310 kg ha^-1^ in 2016 in moisture stress areas (Table 2).

**Table 2:** Maize grain yield response to each nutrient applied in the nutrient omission trials (NOTs) established in 2015 and 2016 seasons

Year	Study sites	Maize yield response (kg ha ^-1^)			
		N	P	K	(S, Mg, Ca, B, Zn)
2015	Bako Tibe	2657	616	-378	-508
	Gobu Sayo	3922	1574	1346	-976
	Omo Nada	4005	685	371	-778
	Kersa	4266	991	163	-224
	Hawassa	1513	-47	-6	505
	Adami Tullu	383	498	-159	586
	Bulbula	562	1104	498	-229
2016	Bako area	3648	349	-695	407
	Jimma area	5454	2056	407	-549
	Hawassa	1500	1320	213	469
	Adami Tullu	3310	1628	411	1496
	Bulbula	2479	1880	-55	-179

Yield response was calculated considering yield from NPK plot as maximum yield and subtracting the yield obtained from missing nutrient (e.g Yield from NPK plot –Yield from PK plot =Yield response due to N)

Maize showed little or no response to N application at eight experimental fields in Bako and Jimma areas during 2015 season (data not presented). At these few sites with nonresponsive soils, the average grain yield for N omitted plots was 7.9 t ha^-1^ compared with an average grain yield of 8.3 t ha^-1^ for the NPK treated plots.

### 3.3 Maize Yield Response to P

During 2015, grain yield on average increased by 967 kg ha^-1^ (range of 616 to 1574 kg ha^-1^) in high rainfall areas and by 801 kg ha^-1^ (range of 498 to 1104 kg ha^-1^) in moisture stress areas due to P application across all experimental sites except at Hawassa. During 2016, grain yield on average increased by 1202 kg ha^-1^ (range of 349 to 2056 kg ha^-1^) in high rainfall areas and by 1609 (range of 1320 to 1880 kg ha^-1^) in moisture stress areas due to P application across all experimental sites (Table 2).

During 2015 season, the highest yield response to P was observed at Gobu Sayo (1574 kg ha^-1^) and Bulbula (1104 kg ha^-1^). During 2016 season, yield responses to P application of 2056, 1880 and 1628 kg ha^-1^ were observed in Jimma area and at Bulbula and Adami Tullu, respectively (Table 2).

### 3.4 Maize Yield Response to K and Other Nutrients

Overall, there was little or no yield response to all other nutrients applied (i.e. potassium, and secondary and micronutrients) (Table 2). However, maize responded to K application (1346 kg ha^-1^) in Gobu Sayo in 2015 season and to secondary and micronutrients (1496 kg ha^-1^) at Adami Tullu in a good rainfall season in 2016 (Table 2).

### 3.5 N and P Uptake

**Fig. 6:**
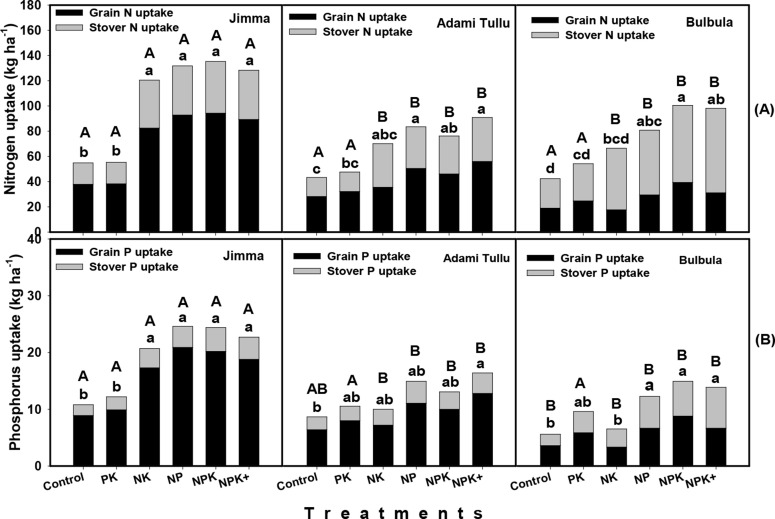
Total plant uptake of nitrogen (A) and phosphorus (B) in Jimma area, Adami Tullu and Bulbula

Total N uptake significantly differed between the high rainfall and moisture stress areas for every same treatment (except for control and PK) as well as between treatments of every same location (Fig. 6A). The total N uptake for every same treatment (except for control and PK) was significantly higher in high rainfall area (Jimma area) than in moisture stress areas (Adami Tullu and Bulbula) (Fig. 6A). Total N uptake, however did not significantly differ between the two moisture stress locations. The average N uptake (for all treatments) by the crop is 1.7-fold higher at high rainfall compared to the moisture stress area. The total N uptake was significantly lower for the control and N omitted treatments compared to other treatments at all experimental sites. In Jimma, the total N uptake ranged from 55 kg ha^-1^ (for the control and N omitted treatments) to 135 kg ha^-1^ (for the NPK treatment). At Adami Tullu and Bulbula, the total N uptake ranged from 38.5 (for control) to 82 kg ha^-1^ (for NPK treatment), and from 40.1 (for control) to 89.2 kg ha^-1^, (for NPK treatment), respectively. In Jimma area and at Adami Tullu, a higher proportion of the total N was taken up by the grain than the stover, while at Bulbula a higher proportion of total N was taken up by the stover than by the grain (Fig. 6A).

Different small letters denote significant difference between treatments at each location whereas; different capital letters denote significant difference between locations for similar treatment

Similar to total N uptake, the total P uptake also significantly differed between the high rainfall and moisture stress areas for every same treatment (except PK) as well as between treatments of same location (Fig. 6B). The total P uptake for every same treatment (except PK) was significantly higher in high rainfall area (Jimma area) than in moisture stress areas (Adami Tullu and Bulbula) (Fig. 6B). The average P uptake (for all treatments) by the crop is 2.3-fold higher at high rainfall compared to the moisture stress areas. The total P uptake for NP, NPK and NPK+ treatments were significantly higher than for the control, N and P omitted plots, especially in Jimma area and at Bulbula. The total P uptake was generally lower for the control and N omitted treatments at Jimma and for the control and P omitted plots at Bulbula (Fig. 6B). In Jimma area, the P uptake ranged from 10.8 kg ha^-1^ (for the control) to 24 kg ha^-1^ (for NP and NPK treatments). However, at the moisture stress areas, P uptake was lower ranging from 6.5 kg ha^-1^ to 12.5 kg ha^-1^ at Adami Tullu and from 4.3 to 11.0 kg ha^-1^ at Bulbula. Conversely to the grain and stover N uptake, the grain P uptake was consistently higher than the stover P uptake for all the three locations (Fig. 6B).

### 3.6 Agronomic Efficiency of N and P

The agronomic efficiency of nitrogen (AE_N_) did not vary between treatments in Jimma area but varied at the moisture stress areas. It also varied between locations for the same treatment (Table 3). The AE_N_ ranged from 24.8 to 32.5 kg grain kg^-1^ N in Jimma area. At the moisture stress areas it ranged from 1.0 kg grain kg^-1^ N (NK treatment) to 10.2 kg grain kg^-1^ N (NPK treatment) at Adami Tullu and from 0.1 kg grain kg^-1^ N (NK treatment) to 8.3 kg grain kg^-1^ N (NPK treatment) at Bulbula. The AE_N_ was significantly lower for the NK treatment compared to the rest of the treatments both at Adami Tullu and Bulbula. On the other hand, for every same treatment, the AE_N_ was significantly higher for the high rainfall than low rainfall areas (Table 3).

The agronomic efficiency of phosphorus (AE_P_) varied between treatments at all experimental sites. In Jimma area, the agronomic efficiency of phosphorus (AE_P_) ranged from 3.3 kg grain kg^-1^ of P (N omitted plot) to 100.8 kg grain kg^-1^ of P (NPK treatment). Compared to the high rainfall area, the AE_P_ in the moisture stress areas was remarkably lower and ranged from 1.4 to 27.6 kg grain kg^-1^ P and from 13.5 kg grain kg^-1^ P to 27.6 kg grain kg^-1^ P at Adami Tullu and Bulbula, respectively (Table 3). The AE_P_ was significantly lower when N was omitted for Jimma area and Adami Tullu. For every same treatment the AE_P_ was also significantly higher for the high rainfall area than for the moisture stress areas, except for the PK treatment where there was no significant difference in AE_P_ (Table 3).

**Table 3:** Agronomic efficiency of nitrogen (N) and phosphorus (P) as affected by fertilizer treatments in major maize production areas in 2015

Treatments	Agronomic efficiency of N (kg grain kg^-1^ N applied)			Agronomic efficiency of P ( kg grain kg^-1^ P applied )		
	Jimma	Adami Tullu	Bulbula	Jimma	Adami Tullu	Bulbula
Control	-	-	-	-	-	-
PK	-	-	-	3.3bA	1.4bA	13.5bA
NK	24.8aA	1.0bB	0.1bB	-	-	-
NP	31.3aA	8.0aB	4.2abB	97.1aA	20.9aB	15.1bB
NPK	32.5aA	8.4aB	8.3aB	100.8aA	16.1aB	27.6aB
NPK+	29.9aA	10.2aB	4.4abB	93aA	27.6aB	15.8bB
LSD	8.4	6.9	8.0	23.45	14.3	11.2

Different small letters and capital letters denote significant difference between treatments for the same location and between locations for the same treatment, respectively

### 3.7 Apparent Recovery Efficiency of N and P

The apparent recovery fraction of N (ARE_N_) did not differ between treatments at the other two locations except at Bulbula but differed between locations for every same treatment (Table 4). At Bulbula, the ARE_N_ ranged from 0.16 (NK treatment) to 0.41 kg N kg^-1^ applied N (NPK treatment) (Table 4). For every same treatment, the ARE_N_ was significantly higher at the high rainfall area compared to moisture stress areas. However, the ARE_N_ did not differ between the two moisture stress areas.

**Table 4:** Apparent recover fraction of applied N and P fertilizer as affected by fertilizer treatments

Treatments	Apparent Recovery Fraction of N (kg N taken up kg^-1^ of N applied)			Apparent Recovery Fraction of P (kg P taken up kg^-1^ of P applied)		
	Jimma	Adami Tullu	Bulbula	Jimma	Adami Tullu	Bulbula
Control	-	-	-	-	-	-
PK	-	-	-	0.04^bA^	0.03^bA^	0.07^bA^
NK	0.54^aA^	0.20^aB^	0.16^bB^	-	-	-
NP	0.63^aA^	0.30^aB^	0.26^abB^	0.35^aA^	0.12^abB^	0.13^abB^
NPK	0.67^aA^	0.26^aB^	0.41^aB^	0.34^aA^	0.09^abB^	0.17^aB^
NPK+	0.61^aA^	0.37^aB^	0.37^aB^	0.30^aA^	0.15^aB^	0.15^abB^
LSD	0.17	0.21	0.198	0.10	0.106	0.09

Different small letters and capital letters denote significant difference between treatments for the same location and between locations for the same treatment, respectively

The apparent recovery fraction of P (ARE_P_) significantly varied between treatments at all locations and also between high rainfall and moisture stress areas for every same treatment except for the PK treatment (Table 4). However, the ARE_P_ did not differ between the two moisture stress areas. In Jimma area, the P recovery fraction ranged from 0.04 kg P kg^-1^ of applied P (for PK treatment) to 0.35 kg P kg^-1^of applied P (for NP treatment). At the moisture stress areas, it ranged from 0.03 kg P kg^-1^ applied P (for PK) to 0.15 kg P kg^-1^ applied P ( for NPK+ treatment) at Adami Tullu and from 0.07 kg P kg^-1^ of applied P (for PK treatment) to 0.15 kg P kg^-1^ of applied P (NPK) at Bulbula.

## 4. DISCUSSION

### 4.1 Grain Yield Response to N Application

The higher grain yield response to nitrogen (N) application than any other nutrients at all study sites clearly shows that N is the most limiting essential plant nutrient for maize intensification in major maize growing areas of Ethiopia and hence needs special attention. The highly magnificent yield response to the application of 120 kg N ha^-1^ in the high rainfall areas compared to the moisture stress areas explains the fact that application of high dose of N fertilizer in moisture stress areas only slightly improves maize productivity. This is because soil moisture being the medium of nutrient transport to the absorbing root (28), plays a key role in influencing crop response to fertilizer application. Integrating soil moisture conservation with fertilizer management could, therefore, be one of the vital strategies to improve maize productivity in moisture stress areas. The higher grain yield response to N application in the high rainfall area compared to the moisture stress areas is in agreement with the findings of [27], who also reported higher magnitude of yield response to nitrogen application under favourable rainfall conditions than unfavourable conditions. The higher grain yield response under favourable rainfall could be attributed to the availability of more available N forms in the soil solutions owing to sufficient soil moisture as well as to the high water flux, both of which increase the mass flow of nitrogen ions to the root surface enhancing N uptake since mass flow rate is a function of both water flux in the root rhizosphere and nutrient concentration in the soil solution [28].

At Gobu Sayo, only 14% of the fields had a soil total N content that is rated as low while at Bako Tibe 66% of the fields had a soil total N content that is rated as low, and yet the yield response to nitrogen application was higher at Gobu Sayo (3922 kg ha^-1^) than at Bako Tibe (2657 kg ha^-1^).

This suggests that total N content of soil might not necessarily reflect availability of nitrogen for plant uptake. However, at Omo Nada, where 87% of fields had a soil total N that is rated as low, the yield response to N application during 2015 was also correspondingly very high (4005 kg ha^-1^).

### 4.2 Grain Yield Response to P Application

Yield response to P was significantly large at Gobu Sayo (1574 kg ha^-1^) and Bulbula (1104 kg ha^-1^) during 2015 season and at Jimma (2056 kg ha^-1^), Bulbula (1880 kg ha^-1^) and Adami Tullu (1628 kg ha^-1^) in 2016 season (Table 2). The yield response to P was quite small although not nil at Bako Tibe and inconsistent between years at Hawassa. The yield response to P during both years was, however, not as high as the yield response to N application and such lesser yield response to P application can be attributed to P fixing nature of the weathered nitisols and calcareous soils of the high and low rainfall areas, respectively. It may also be due to the carryover effects of previous P fertilizer application, especially in some sites where the available P before planting was already in the high range (Table 1).

Grain yield response to application of 40 kg ha^-1^ P was remarkably higher at Gobu Sayo and Bulbula compared to the other experimental sites during 2015, while the response was higher for all experimental sites except for Bako Tibe during 2016. This differential yield response to P application across the experimental sites may only partly and not fully be explained by the difference in the levels of available soil P across the locations. In the high rainfall areas such as Gobu Sayo, none of the fields (Table 1) had available soil P that is above the critical P for maize (12-17 mg kg^-1^ soil) suggested by [29], which can explain the higher yield response to P application (Table 2). At Bako Tibe, however, only 19% of the fields had available soil P content that was greater than the critical soil P of 12-17 mg kg^-1^ soil [29] and yet yield response to P application was lower (Table 2) perhaps due to the P fixation as a result of acidity. At Omo Nada and Kersa, 40% and 44% of the fields had available soil P content that was greater than the critical soil P of 12-17 mg kg^-1^ soil [29] and consequently the yield response to P application was lower compared to Gobu Sayo (Table 2) perhaps due to the carryover effect of previously applied P in most farms.

However, in the moisture stress areas such as Bulbula, Hawassa and Adami Tullu, the soil available P were above the critical level of 12-17 mg kg^-1^ soil in almost all the fields, and yet there was a grain yield response to P application. Thus, under these conditions, the amount of available P in the soil in relation to the critical level cannot explain the yield response to P application. In those moisture stress areas, rather only a small fraction of the available P goes to the soil solution and hence transport to the root surface via diffusion is highly constrained due to both limited water availability and lower nutrient concentration in the soil solution since the rate of diffusion depends on both water availability in the root rhizosphere and the concentration of the nutrient ions in the soil solution [28]. Thus, more P needs to be applied to compensate for the soil water limitation.

### 4.3 Grain Yield Response to K Application

Crop response to K application was limited to few sites unlike the response to N and P application. At Gobu Sayo, only one field had a soil K content that was less than the critical soil K level (234 to 312 mg kg^-1^ soil) suggested by Adeoye and Agboola [30], for maize production and yet there was an average grain yield response of 1346 kg ha^-1^ to potassium application (Table 2). At Omo Nada and Kersa, all the fields had soil potassium content above the critical level suggested by Adeoye and Agboola [30] for maize and consequently there was no remarkable grain yield response to K application at these experimental sites. Surprisingly, at Hawassa, nearly all the fields (94%) had soil K content that were below the critical soil K content for maize production and yet there was no yield response to K application. This could probably be due to dependency of the critical K levels on soil types [31] and thus Hawassa could have lower critical K level than the critical level described by Kihara et al. [10], Adeoye and Agboola [30] for soils in Nigeria. Even within the same country, critical nutrient levels for a crop could vary with locations/regions [31,32]. At Bulbula, 83% of the fields had a soil K contents that were below the critical K of 234 -312 mg kg^-1^ soil for maize production and consequently, there was a grain yield response of 498 kg ha^-1^ to the application of 40 kg ha^-1^ K (Table 2). Unlike 2015 season, where there was no yield response to K application in most sites, except at Gobu Sayo and Bulbula, there was a tendency of yield response to K application in the other sites as well during 2016 season. However, the magnitude of increase in grain yield due to the application of 40 kg K ha^-1^ was smaller ranging only from 213 to 411 kg ha^-1^.

### 4.4 Grain Yield Response to Secondary (S, Mg, Ca) and Micronutrients (Zn, B)

The remarkable grain yield response to application of secondary (S, Ca, Mg) and micronutrients (Zn, B) at Adami Tullu, cannot be attributed to any single nutrient effect as they were applied to the plots altogether. However, analysis of the pre-planting soil samples taken from the experimental fields showed that the soil S content (data not shown) were above the critical soil sulphur level of 10 mg kg^-1^ soil for all fields at Adami Tullu, which may confirm that the grain yield response is less likely due to S application. The soil Ca and Mg content in these two experimental sites were in the sufficient range of 151-350 mg kg^-1^ soil according to rating by Jones [33] for Mg and 1200-2500 mg kg^-1^ soil for Ca, suggesting that the grain yield response might not be related to the application of Ca and Mg containing fertilizers. Moreover, [34], reported that maize grain yields remained unaffected under a wide range of Mg levels with a Ca/Mg ratio ranging between 1.8 to 36.9, suggesting that grain yield response cannot be expected due to Mg application with the narrow Ca/Mg ratio ranging between 6.8 (at Bulbula) and 13.0 (at Hawassa). Thus, the grain yield response could be due to Zn and B application since the Zn contents of all NOTs fields at Bulbula, Hawassa and Adami Tullu were below the critical level of 5-10 mg kg^-1^ soil suggested by [30] or 1.5 mg kg^-1^ soil suggested by Horneck et al. [35] and Jones [33] for maize production. The Zn contents of all NOTs fields in the high rainfall areas were, however, in the high to very high range (Table 1) according to Jones [33] rating and thus response could not be expected. The grain yield response could also be due to B, besides Zn. However there is need for a further study to understand the impact of each of the secondary and micronutrients on maize productivity in those locations where response to combined application of these nutrients were noticed.

### 4.5 N and P Uptake

**Fig. 7:**
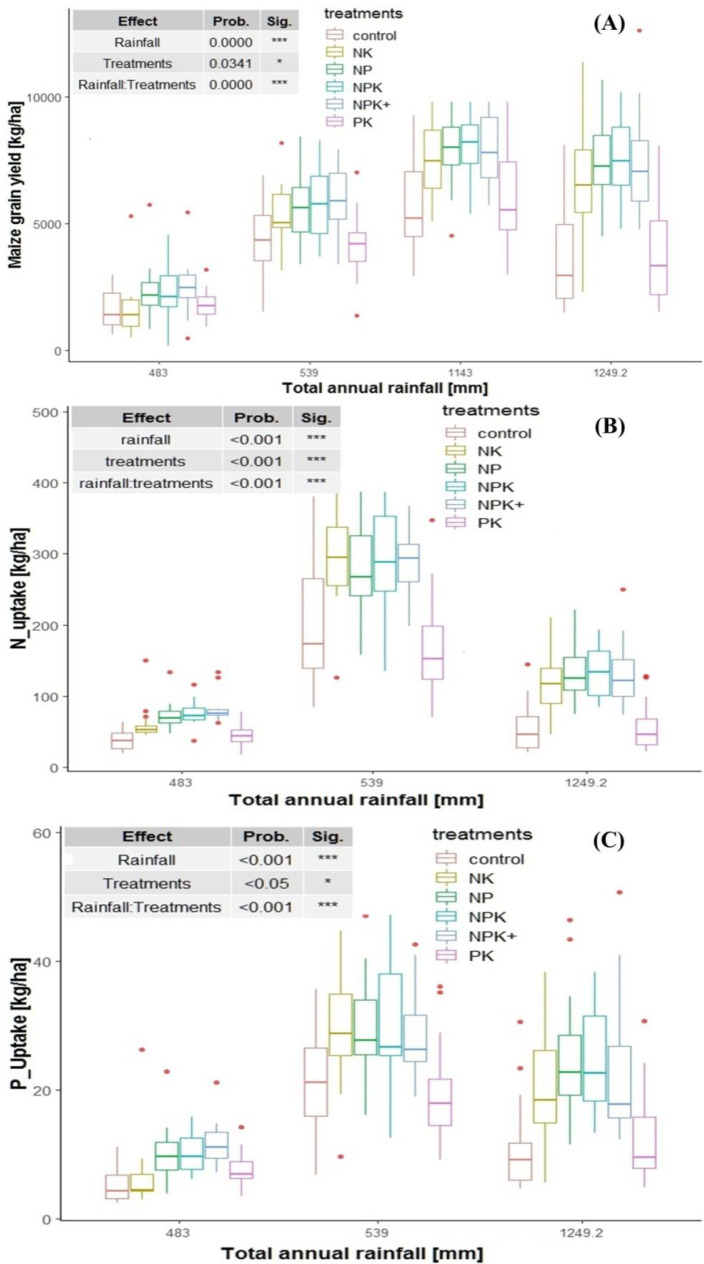
Relationship between total growing season rainfall, grain yield and total N and P uptake

Both N and P uptakes significantly differed between locations for similar treatment and between treatments for same location (Fig. 6A, B). The difference in N and P uptake between locations for every same treatment was attributed to difference in the rainfall amount received during growth periods (Fig. 7). The N and P uptakes were higher in the high rainfall area than in the moisture stress areas. The total N uptake for N applied treatments were on average more than 2-fold higher than that of the control and N omitted treatments (Fig. 6A) in Jimma area, while it was less than 2-fold at Adami Tullu and Bulbula suggesting that the N uptake efficiency was lower in the moisture stress areas. This is further supported by the lower N recovery efficiency observed for the moisture stress experimental sites (Table 4). Likewise, the total P uptake by the crop in Jimma area was more than 2-fold higher than that of Adami Tullu and Bulbula. The average total P uptake for the P applied treatments was only slightly higher than that of the P omitted treatment but more than 2-fold higher than that of the control and N omitted treatments (Fig. 6B) in Jimma area. This suggests that under favourable rainfall, the indigenous soil P can be sufficient to support crop growth if N is not limiting in the soil. However, at the moisture stress experimental sites (Adami Tullu and Bulbula), the average P uptake for all the P applied treatments was only 1.5-fold higher than the control, N and P omitted plots. In those locations, P uptake was highly constrained especially when, P fertilizer was omitted. When sufficient P is not applied, the application of N fertilizer alone cannot support crop growth due to limited availability of indigenous soil P in the soil solution owing to moisture stress and consequently affecting P transport to the root surface for plant uptake. Thus, the P uptake efficiency becomes lower in the moisture stress areas compared to the high rainfall counterparts.

The lower total N and P uptake per hectare in the moisture stress areas compared to the high rainfall areas like Jimma area, is related mainly to both lower grain and biomass yields than to the difference in nutrient concentration in the grain and strover, since grain N concentration was even higher for the moisture stress areas than for Jimma area (data not shown). The total N and P uptake by the crop (kg ha^-1^) in the current study was lower than the N and P uptake by maize under different fertilizer treatments reported by Bąk et al. [36]. On the other hand, the total N and P uptake values reported by Kurwakumire et al. [11] for maize was slightly lower than what was observed for the high rainfall area (Jimma area) but is comparable with what is recorded for the moisture stress areas in the current study. Although nutrient efficiency includes both uptake and utilization efficiency components, this study focused on exploring only the uptake efficiency component specifically the agronomic efficiency and apparent recovery efficiency since physiological efficiency is varietal character which cannot easily be improved through agronomic intervention unlike the uptake efficiency.

### 4.6 Agronomic Efficiency of N and P

The agronomic efficiency of nitrogen (AE_N_) highly contrasted between the high rainfall and moisture stress areas. Significantly higher AE_N_ was observed for the high rainfall area than for the moisture stress areas for every same treatment (Table 3). For instance, the agronomic efficiency of N for the same treatment was 4-fold higher in Jimma area than Adami Tullu/Bulbula. The agronomic efficiency of N reported in the current study for the NPK treatment for high rainfall area is very close to the agronomic efficiency of N reported by Kurwakumire et al. [11], which was 29-35 kg grain kg^-1^ N for the NPS applied treatment and 31-36 kg grain kg^-1^ N for the NPKS applied treatments for different field types.

A similar trend to AE_N_ was observed with the agronomic efficiency of phosphorus (AE_P_), in that AE_P_ also significantly differed between high rainfall and moisture stress areas (Table 3). In the high rainfall area, the maximum agronomic efficiency of P was 100.8 kg grain kg^-1^ of P (for NPK treatment), while in the moisture stress areas, it was lower (27.6 kg grain kg^-1^ P) for NPK treatment at Bulbula and for NPK+ at Adami Tullu (Table 3). On average, AE_P_ was more than 3.5-fold higher for the high rainfall area compared to moisture stress areas. The agronomic efficiency of P also varied between treatments at both contrasting agro-ecologies but with different magnitude. Omission of N (i.e. PK treatment) highly reduced AE_P_, suggesting that P application in the absence of N cannot improve the agronomic efficiency of P. The absence of N application reduced agronomic efficiency of P from 100.8 to 3.3 kg grain kg^-1^ P applied in Jimma area and from 16.1 to 1.4 kg grain kg^-1^ P applied at Adami Tullu and from 17.6 to 13.5 kg grain kg^-1^ P applied at Bulbula. The maximum agronomic efficiency of P reported by Kurwakumire et al. [11] ranged between 50 and 52 kg grain kg^-1^ P for optimum fertilizer level and this was lower than the highest AE_P_ we recorded for the high rainfall areas (100.8 kg grain kg^-1^ of P) but higher than the highest AE_P_ we recorded for the moisture stress areas (27.6 kg grain kg^-1^ P) in the current study. Improving the agronomic efficiency is a core objective of any agronomist, to enable farmers to obtain higher profits. Selecting balanced fertilizer combination that confers the highest agronomic efficiency of each nutrient is quite important since the findings from this study as well as the findings of Kurwakumire et al. [11] confirm this concept. The coapplication of N and P is especially very important since absence of one of these nutrients remarkably reduce the agronomic efficiency of the other nutrient as observed in the current study.

### 4.7 Apparent Recovery Efficiency of N and P

**Fig. 8:**
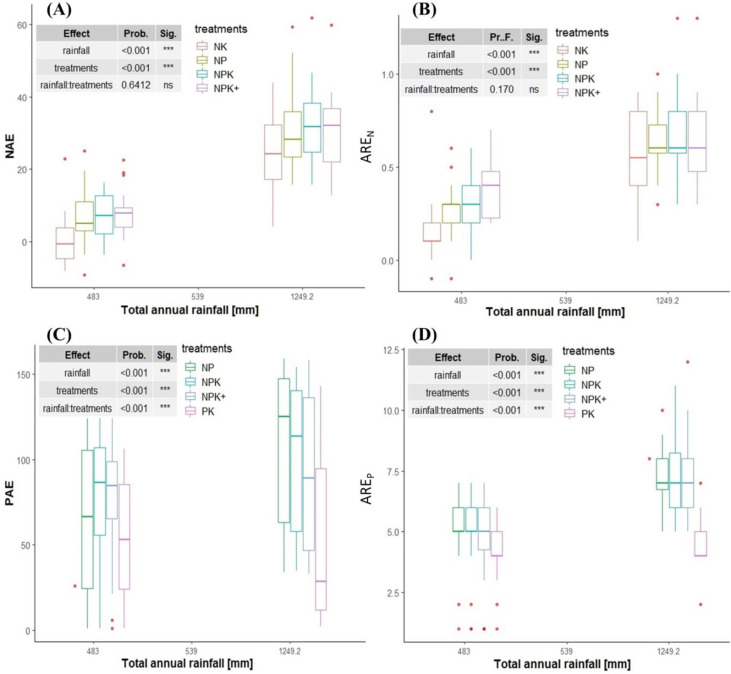
Relationship between total growing season rainfall, agronomic efficiency of N and P and apparent recovery fraction of N and P

The apparent recovery fraction of N (ARE_N_) did not significantly differ between treatments except for Bulbula but differed between locations for every same treatment (Table 4). The ARE_N_ was 1.7-fold higher for high rainfall area compared to moisture stress areas under the application of balanced NPK fertilizer and this was related to sufficiency of rainfall since growing season rainfall amount was the most important variable that influenced fertilizer recovery efficiency between the two contrasting agro-ecologies (Fig. 8B and 8D).

Our study showed that with the application of balanced NPK fertilizer, up to 67% of the applied N fertilizer could be recovered by maize crop in the high rainfall area while only up to 37/ 41% of the applied N fertilizer could be recovered by maize crop in the two moisture stress areas, Adami Tullu/Bulbula (Table 4). Kurwakumire et al. [11] also observed different recovery efficiencies of N at different locations, which was also affected by fertilizer treatments, unlike our finding. They observed higher N recovery fraction of 0.79 and 0.83 kg N kg^-1^ of applied N, with the application of balanced NPS and NPKS nutrients, respectively compared to the application of NK alone, where the ARE_N_ was only 0.44, at similar locations. Thus, their finding supports the findings we observed in the current study.

The apparent P recovery efficiency (ARE_P_) significantly differed between locations for every same treatment as well as between treatments for the same locations (Table 4). In the high rainfall area, the maximum ARE_P_ observed was 0.35 kg P kg^-1^ of applied P (NP and NPK treatments) (Table 4). However, the maximum possible ARE_P_ was only 0.17 kg P kg^-1^ of applied P (for NPK treatment) and 0.15 kg P kg^-1^ of applied P (for NPK+) at the moisture stress locations (Bulbula and Adami Tullu, respectively). Thus, in the moisture stress areas maize crop could only recover up to 17% of the P fertilizer applied (only half the amount recovered in the high rainfall area), while maize crops in the moisture sufficient areas could recover up to 35% of the P fertilizer applied under balanced NPK fertilization. The low P recovery efficiency in the moisture stress areas can be related to insufficient soil moisture which brings about low P diffusion rate to the root surface [28] than to the soil pH, which also usually affects P recovery efficiency. The P recovery efficiency was very low for the treatments were N was missing in the current study (Table 4). This indicates that the co-application of N with other nutrients enhances the P recovery efficiency, as was also reported by Kurwakumire et al. [11]. The P recovery efficiency reported by Kurwakumire et al. [11] was equivalent to the P recovery efficiency observed for the moisture stress areas but lower than that of the high rainfall areas. In a nutshell, the lower agronomic as well as apparent recovery efficiencies of both N and P, in the moisture stress areas compared to the moisture sufficient areas was mainly related to difference in the amount of total growing season rainfall in the two agro-ecosystems as can be realized from the strong positive effect of growing season rainfall on both Agronomic and apparent recovery efficiencies of N and P (Figs. 8A, B, C, D).

## 5. CONCLUSIONS

High degree of variability in maize response to fertilizer application was observed between the different study sites denoted as contrasting agro-ecologies (i.e high rainfall and moisture stress areas). Response to fertilizer application in terms grain yield, nutrient uptake, agronomic and apparent recovery efficiencies of N and P was higher in high rainfall than low rainfall areas, as growing season rainfall amount was the determinant of the variability. Nitrogen was the most yields limiting in almost all study sites while P was the second most yield limiting in some study sites. The responses of maize to potassium and secondary and micronutrients were highly localized; potassium was important at Gobu Sayo while micronutrients were important at Adami Tullu. Thus, application of potassium fertilizer and micronutrients blended fertilizers would be important in such areas as Gobu Sayo and Adami Tullu, respectively. The wide variability in maize yield response to application of different nutrients observed in this study suggests that site-specific nutrient management is fundamental to intensify maize production and productivity. This study has demonstrated that balanced application of nutrients, especially NP and NPK significantly improved nutrient uptake by crop, agronomic and fertilizer recovery efficiencies, regardless of the study sites. The remarkable difference in N and P uptake, N and P agronomic as well as recovery efficiencies between the high rainfall and moisture stress areas implies that soil moisture play a key role in improving nutrient availability in the soil rhizosphere thereby enhancing the agronomic and recovery efficiencies of nutrients through enhancing nutrients concentration in the soil solution as well as their transport to the root surface. Ensuring moisture availability during both side dressing and top dressing of fertilizers is, therefore, very important to optimize the recovery of applied nutrients and minimize nutrient losses to the environment. Mechanisms of improving nutrient efficiencies such as moisture conservation options through tideridging, practicing supplementary irrigation when possible should be sought in moisture stress areas. Proper management of N fertilizer is vital for increasing maize yields. Thus, policies that promote farmers’ access to N fertilizers are critical for intensification of maize productivity in Ethiopia.
